# Supplementary material for: Sparsey™: event recognition via deep hierarchical sparse distributed codes
Source: Front Comput Neurosci. 2014 Dec 15;8:160. doi: 10.3389/fncom.2014.00160 (PMC4266026; doi:10.3389/fncom.2014.00160)
Supplement: Supplementary file 1 [file Presentation1.PPTX]

## Slide 1
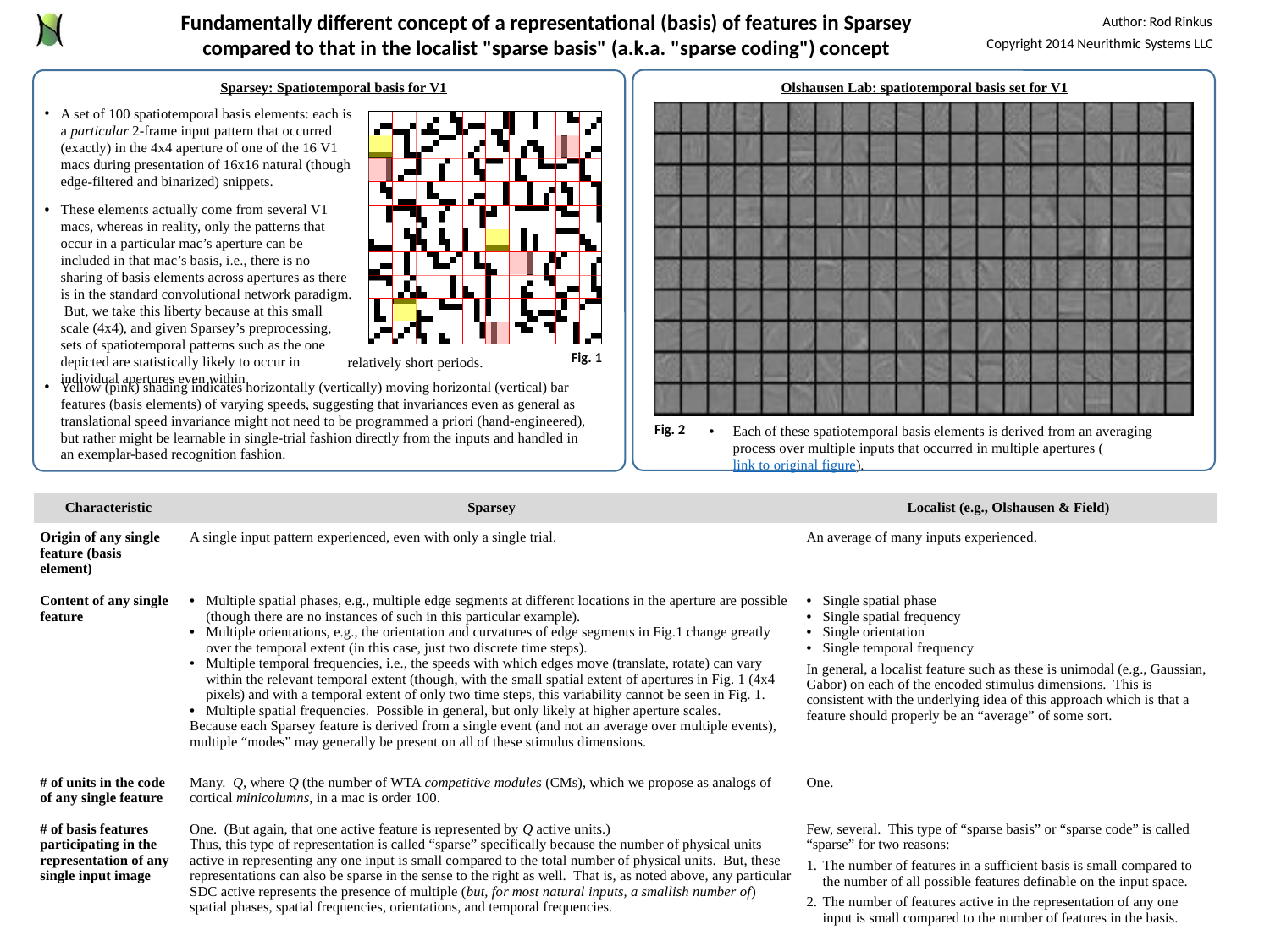

Fundamentally different concept of a representational (basis) of features in Sparsey compared to that in the localist "sparse basis" (a.k.a. "sparse coding") concept
Author: Rod Rinkus
Copyright 2014 Neurithmic Systems LLC
Sparsey: Spatiotemporal basis for V1
Olshausen Lab: spatiotemporal basis set for V1
A set of 100 spatiotemporal basis elements: each is a particular 2-frame input pattern that occurred (exactly) in the 4x4 aperture of one of the 16 V1 macs during presentation of 16x16 natural (though edge-filtered and binarized) snippets.
These elements actually come from several V1 macs, whereas in reality, only the patterns that occur in a particular mac’s aperture can be included in that mac’s basis, i.e., there is no sharing of basis elements across apertures as there is in the standard convolutional network paradigm. But, we take this liberty because at this small scale (4x4), and given Sparsey’s preprocessing, sets of spatiotemporal patterns such as the one depicted are statistically likely to occur in individual apertures even within
relatively short periods.
Fig. 1
Yellow (pink) shading indicates horizontally (vertically) moving horizontal (vertical) bar features (basis elements) of varying speeds, suggesting that invariances even as general as translational speed invariance might not need to be programmed a priori (hand-engineered), but rather might be learnable in single-trial fashion directly from the inputs and handled in an exemplar-based recognition fashion.
Fig. 2
Each of these spatiotemporal basis elements is derived from an averaging process over multiple inputs that occurred in multiple apertures (link to original figure).
| Characteristic | Sparsey | Localist (e.g., Olshausen & Field) |
| --- | --- | --- |
| Origin of any single feature (basis element) | A single input pattern experienced, even with only a single trial. | An average of many inputs experienced. |
| Content of any single feature | Multiple spatial phases, e.g., multiple edge segments at different locations in the aperture are possible (though there are no instances of such in this particular example). Multiple orientations, e.g., the orientation and curvatures of edge segments in Fig.1 change greatly over the temporal extent (in this case, just two discrete time steps). Multiple temporal frequencies, i.e., the speeds with which edges move (translate, rotate) can vary within the relevant temporal extent (though, with the small spatial extent of apertures in Fig. 1 (4x4 pixels) and with a temporal extent of only two time steps, this variability cannot be seen in Fig. 1. Multiple spatial frequencies. Possible in general, but only likely at higher aperture scales. Because each Sparsey feature is derived from a single event (and not an average over multiple events), multiple “modes” may generally be present on all of these stimulus dimensions. | Single spatial phase Single spatial frequency Single orientation Single temporal frequency In general, a localist feature such as these is unimodal (e.g., Gaussian, Gabor) on each of the encoded stimulus dimensions. This is consistent with the underlying idea of this approach which is that a feature should properly be an “average” of some sort. |
| # of units in the code of any single feature | Many. Q, where Q (the number of WTA competitive modules (CMs), which we propose as analogs of cortical minicolumns, in a mac is order 100. | One. |
| # of basis features participating in the representation of any single input image | One. (But again, that one active feature is represented by Q active units.) Thus, this type of representation is called “sparse” specifically because the number of physical units active in representing any one input is small compared to the total number of physical units. But, these representations can also be sparse in the sense to the right as well. That is, as noted above, any particular SDC active represents the presence of multiple (but, for most natural inputs, a smallish number of) spatial phases, spatial frequencies, orientations, and temporal frequencies. | Few, several. This type of “sparse basis” or “sparse code” is called “sparse” for two reasons: The number of features in a sufficient basis is small compared to the number of all possible features definable on the input space. The number of features active in the representation of any one input is small compared to the number of features in the basis. |
